# Color Change after 25% Hydrogen Peroxide Bleaching with Photoactivation: A Methodological Assessment Using Spectrophotometer versus Digital Photographs

**DOI:** 10.3390/ma15145045

**Published:** 2022-07-20

**Authors:** Muhittin Ugurlu, Nadin Al-Haj Husain, Mutlu Özcan

**Affiliations:** 1 Department of Restorative Dentistry, Faculty of Dentistry, Süleyman Demirel University, 32200 Isparta, Turkey; 2 Department of Reconstructive Dentistry and Gerodontology, School of Dental Medicine, University of Bern, 3010 Bern, Switzerland; nalhaj88@gmail.com; 3 Division of Dental Biomaterials, Clinic of Reconstructive Dentistry, Center of Dental Medicine, University of Zurich, 8032 Zurich, Switzerland; mutlu.ozcan@zzm.uzh.ch

**Keywords:** color stability, dental photography, retrospective study, spectrophotometer, tooth whitening

## Abstract

This study aimed to evaluate the color change of teeth bleached with light activation using two different objective color measurement approaches after two years of clinical follow-up. A cross-sectional retrospective clinical study according to STROBE was followed including 30 participants. The 25% hydrogen peroxide gel (Philips Zoom) was applied with a supplementary LED light for 15 min in four cycles. Tooth color was assessed based on CIEL*a*b* values using a spectrophotometer (Spectroshade) at different time points (baseline, post bleaching, 1 week, 1 year, and 2 years). Standardized digital photographs were taken at each time point. The L*, a*, and b* values were measured from the digital photographs using Adobe Photoshop software. The color difference (ΔE) was separately calculated using the L*, a*, and b* values obtained with spectrophotometric and photographic analyses at each evaluation time. Data were analyzed with non-parametric tests (*p* < 0.05). A color regression was detected by both measurement approaches after 1 and 2 years (*p* < 0.05). Greater ΔE values were acquired with the spectrophotometer compared to the digital photographic analysis (*p* < 0.05). Although a greater color change was observed with the spectrophotometer, both approaches were able to detect the color rebound using the 25% hydrogen peroxide light-activated in-office system. Digital photographic analysis might therefore be used to assess color change after bleaching.

## 1. Introduction

The demand from patients for tooth bleaching is ever-increasing in the treatment of tooth discolorations [1]. Discolored teeth may be treated by vital and non-vital bleaching techniques. At-home and in-office bleaching is used for the whitening of discolored vital teeth in dental practice [1,2]. At-home bleaching has some disadvantages related to the patient′s ability to use bleaching trays and compliance and needs closer monitoring in clinical scenarios, where extensive tissue recession or deep unrestored abfraction lesions are present [2]. In-office bleaching allows close dentist control, avoidance of soft-tissue exposure and material ingestion, reduced total treatment time, and greater potential for immediate results, enhancing patient satisfaction and motivation and minimizing complications of the use of bleaching trays [3]. Commonly used products are composed of percarbamide, peroxyacetic acid, sodium perborate, light-activated riboflavin and/or riboflavin derivatives, perborate/H_2_O_2_ mixtures, and sodium percarbonates. Hydrogen peroxide (H_2_O_2_) is a bleaching agent, which is the active molecule that acts as a strong oxidizing agent through the formation of free radicals, reactive oxygen molecules, and perhydroxyl anions [2]. Although the mechanism of bleaching using hydrogen peroxide is not entirely known, it is believed that the peroxide reacts with the organic chromophores, which cause discoloration in teeth after it is diffused into enamel and dentin [2]. Hydrogen peroxide is usually employed at approximately 35% concentrations for in-office bleaching, but it can be used in different concentrations [3,4]. However, the contact of hydrogen peroxide with soft tissues and tooth sensitivity may be complications during the In-Office method.

The use of a light source has been recommended to hasten the action of the bleaching agent [5]. It has been stated that light activation might accelerate in-office bleaching with a lower concentration of hydrogen peroxide by enhancing the dissociation rate of hydrogen peroxide, thus reducing the time that is required for the bleaching protocol to occur [4,6,7]. The bleaching gels involve coloring agents or pigments that improve the interaction of visible light, promoting absorption of the light and conversion into heat. The light source influences the bleaching gel by increasing the temperature of the hydrogen peroxide, therefore accelerating the reaction of forming hydroxyl and oxygen radicals. Nevertheless, it has been reported that the light-activated bleaching improved the bleaching efficacy for a short period, but it did not impact long-term results [8]. Tooth bleaching is an effective treatment method that meets the esthetic expectations of patients. However, the stability of the bleaching treatment results over time is also a major concern for patients and clinicians [8]. Previous clinical follow-up studies have assessed the color stability obtained by different tooth-bleaching products, concentrations, and application protocols, although most of them only evaluate color stability over short periods [4,5,8,9,10,11,12]. Long-term follow-up studies are much more important [9]. There are also long-term clinical trials, but the number of them is few [7,13,14,15].

The assessment of tooth color in clinical practices is of great importance for the esthetic result of any dental treatment [8]. The tooth color and the results of bleaching can be measured by subjective methods and objective instruments [13]. Color evaluation with subjective methods is quite hard in clinical practices since several parameters may affect the color, such as daylight, ambient light, ambient conditions, and patient-related factors (such as age, gender, baseline tooth color, or the patient’s sitting position and use of lipstick) [2]. The use of instrumental methods, such as spectrophotometers and digital cameras, facilitates color evaluation and presents more accurate and objective results [7,16,17]. Spectrophotometry is a frequently used instrumental approach in studies for the assessment of tooth color, but it has some disadvantages [18]. Fogging may occur in the photos taken by the spectrophotometer, which might lead to inaccurate readings [8]. The measurement of translucency, which is an inherent property of teeth, is also difficult because the curved surface of the tooth may negatively affect the uniform reflectance of light to the spectrophotometer [18]. It has been reported that an imaging system might be a reliable alternative color measurement method to the spectrophotometer [19]. Nowadays, spectrophotometers are still commonly employed in clinical practice, while the use of digital photography is considered more, yet not regularly used [20,21,22,23,24]. It has been stated that measuring systems have become established that describe the tooth shade based on the remission spectrum by digital photographic analysis and image editing software, which is a simple, easily available, and cheap method [5,25,26]. However, evaluation of the color change of bleached teeth by spectrophotometric and digital photographic analyses in a clinical setup has not yet been compared.

Therefore, the objective of this study was to assess the stability of tooth color after two years of follow-up in patients treated by a 25% H_2_O_2_ light-activated office bleaching system using two different instrumental color measurement methods, including a spectrophotometer and digital photographs. The null hypotheses were (1) that there would be no significant color rebound at different time points and (2) that there would be no significant difference between color change values evaluated by spectrophotometric and digital photographic analyses.

## 2. Material and Methods

The cross-sectional retrospective study was approved by the Institutional Ethical Committee (registration number: 2020/74). Data were compiled from patients treated at the Department of Restorative Dentistry at the Suleyman Demirel University. In this retrospective study, the patient record files of 30 patients were used. The patients were treated for bleaching and visited the clinical practice for check-up visits 1 week, 1 year, and 2 years after the end of the treatment. This study was prepared according to the strobe guideline. The following inclusion criteria were applied: Patients (>18 years) with good general oral health who were caries-free without restorations at the labial surfaces and treated in the mentioned unit between March 2016 and March 2018; the use of 25% hydrogen peroxide gel (Zoom2, Discus Dental, Ontario, CA, USA) with a supplementary LED light (Zoom WhiteSpeed, Discus Dental, Ontario, CA, USA) over a period of 15-min cycles for a total exposure time of 60 min; the availability of spectrophotometric color registries and standardized intraoral digital photographs at all the study time points (before treatment, immediately after treatment, and 1 week, 1 year, and 2 years after the end of treatment).

Subjects were excluded in cases where they had undergone previous tooth whitening procedures, were pregnant, and had severe internal tooth discoloration or labial restorations.

The chosen time points were determined based on previous studies [7,14]. Before starting the in-office whitening technique, the teeth were pumiced, and the gingival tissue was isolated using a light-cured resin dam (Liquidam, Discus Dental, Stamford, CT, USA). The 25% hydrogen peroxide gel was applied to the teeth. Visible light irradiation was applied with the light-curing unit for 15 min. Then, the gel was removed with gauze and the procedure was repeated four times. All the bleached teeth were vital. A total of 30 patients subjected to tooth bleaching treatment were selected, and a study sample of 30 participants was established following the application of the inclusion criteria and available patient population.

**Figure 1:**
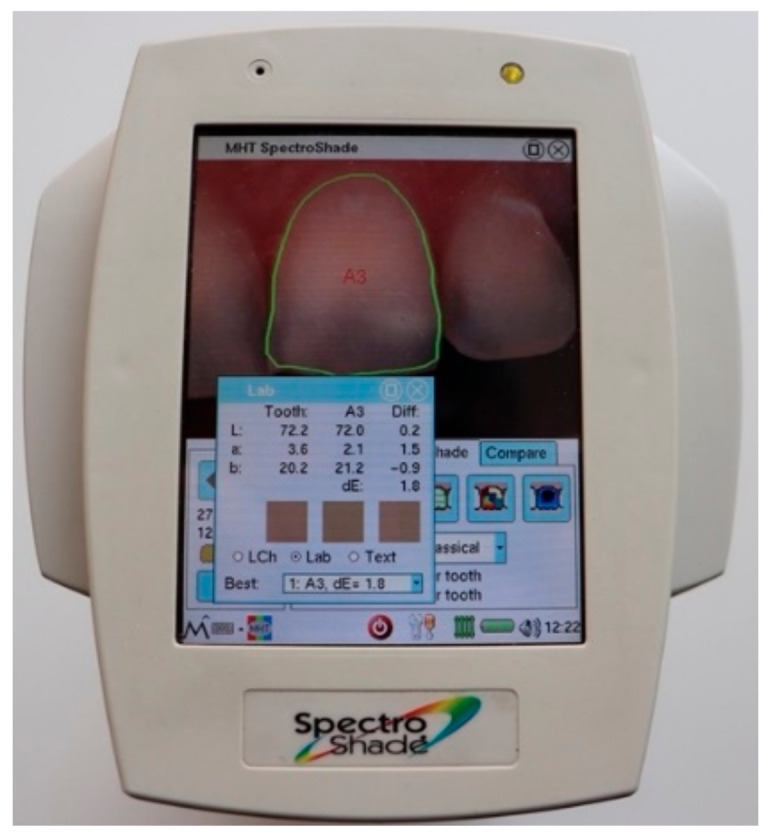
Defining the tooth area and the measurement of CIEL*a*b* values by the spectrophotometer.

The spectrophotometric evaluation and photographs were performed under standardized daylight, ambient light, and ambient conditions. An intraoral spectrophotometer (Spectroshade, MHT Optic Research AG, Niederhasli, Switzerland) was used for the color assessment based on the CIE (Commission International de l’Eclairage) L*a*b* system during the different evaluation times (baseline, after bleaching, 1 week, 1 year, 2 years). Before each measurement, the spectrophotometer was calibrated, and the camera’s white balance was adjusted. The mouthpiece was carefully positioned over the tooth required. The correct and desired position of the tooth was verified by a horizontal green line, and the image was recorded. The software automatically outlined the teeth, and the CIE L*a*b* coordinates were determined (Figure 1). The measurements were performed by the same operator, and the position of the measuring probe was reproducibly standardized for all subsequent measurements. The six anterior maxillary teeth were evaluated from each patient for this study, as in previous studies [4,5,7].

**Figure 2:**
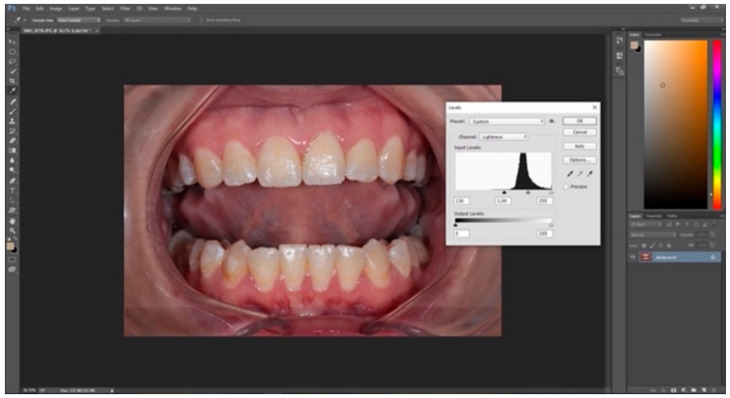
The selection of the tooth to be measured, and the metering of the L* value through the software.

The standardized digital intraoral photographs were taken by a DSLR camera (Canon EOS 800 D; Canon, Tokyo, Japan) at the time points. An image-stabilizing macro lens (Canon EF 100 mm; Canon, Tokyo, Japan) and a macro twin flash (YN24EX; Yongnuo, Shenzhen, China) were used during the photoshoot. The settings on the camera were manually set, and the flash unit was automatically adjusted. All measurements were performed under standardized ambient conditions to guarantee accuracy, stability, and reproducibility. At every stage, the patient′s position and the distance of the patient from the lens were maintained. The L*, a*, b* values were obtained from the digital photographs using software (Adobe Photoshop; Adobe Systems, San Jose, CA, USA), as shown in Figure 2. The photoshop L*, a*, b* values were transformed into the CIE L*, a*, b* values, as in a previous study [27,28].

The range of these values was different in both systems [28]. In photoshop, the range of the mean L* value was 0 to 255. The CIE L* value ranged from 0 to 100. This transformation was performed by using the following formula: CIE L* = L*(photoshop) × 100/255. The a* and b* values were transformed in the same manner. The photoshop values ranged from 0 to 255, and the CIE a*, b* values from +120 to +120. This transformation was performed by using the following formula: CIE a* = [a*(photoshop) − 128] 240/255, CIE b* = [b*(photoshop) − 128] × 240/255 [28].

Based on the CIE L*, a*, b* values obtained with spectrophotometric and photographic analyses, the color difference (ΔE) was separately calculated at each study time point with respect to the baseline using the following formula: ΔE = [(ΔL*)^2^ + (Δa*)^2^ + (Δb*)^2^]^1/2^. Upon completion of the bleaching treatment, and 1 week, 1 year, and 2 years after treatment, the patients were asked if they were satisfied with their teeth colors using the scale: none, moderate, and a lot.

The data were analyzed with a statistical program (SPSS Vers 20, Chicago, IL, USA). The values of ΔL, Δa, Δb, and ΔE calculated at different test periods were assessed. The test of the data for normal distribution was performed by the Kolmogorov–Smirnov test. The data obtained by different measurement methods at the same time point was compared via the Mann–Whitney test. The analysis of data acquired by the same measurement method at different evaluation times was performed using the Friedman test. Pairwise comparisons were made with the Wilcoxon test. The statistical significance level was set at 0.05 for all analyses.

## 3. Results

The age of the included patients ranged between 22 and 40 (mean ± SD: 28.40 ± 5.68 years). Mean ± SD of the parameters L*, a*, and b* and the ΔL, Δa, Δb, and ΔE values obtained with spectrophotometric and digital photographic analyses are shown in Table 1.

**Figure 3:**
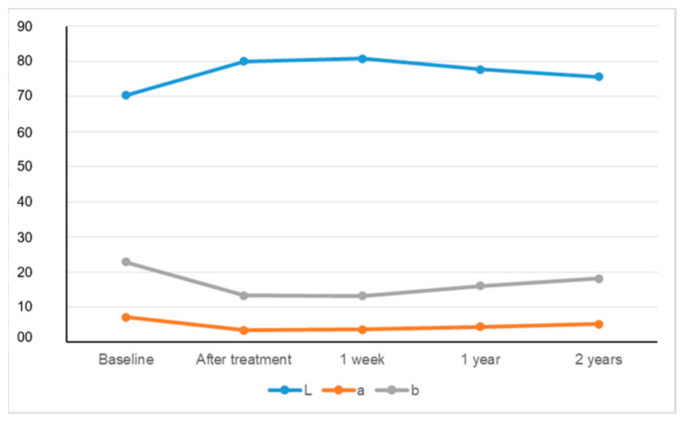
The change of CIEL*a*b* values measured by spectrophotometer over time. *Y-axis defines the numeric values of the parameters L, a, and b.

**Figure 4:**
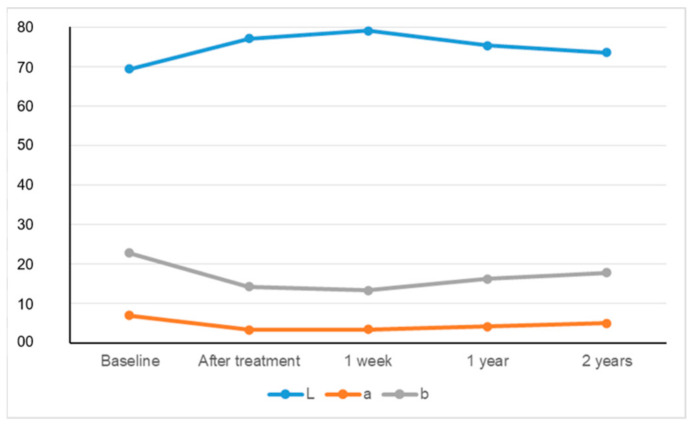
The change of CIEL*a*b* values measured by digital photographs and software over time. *Y-axis defines the numeric values of the parameters L, a, and b.

The means of ΔL, Δa, Δb, and ΔE values, including standard deviations, are given with a statistical comparison of evaluation times and measurement approaches in Table 1 The changes in L, a, and b values over time are graphically presented in Figure 3 and Figure 4.

The L* values increased up to 1 week, and then decreased. The a* values reduced immediately after treatment, and then increased. The b* values diminished up to 1 week and were then enhanced. The ΔE values increased up to 1 week following bleaching and then decreased (*p* < 0.05). The measurement methods both showed effectiveness regarding the bleaching technique, but the spectrophotometric evaluation revealed a greater color change compared with the photographic evaluation (*p* < 0.05). The color rebound was observed according to both measurement approaches (*p* < 0.05). Lower ΔE values were obtained with digital photographic analyses than spectrophotometric analyses at all evaluation periods (*p* < 0.05). All patients were very satisfied with their teeth color during all evaluation periods.

## 4. Discussion

The objective of the present clinical study was to evaluate the color relapse of the light-activated in-office bleaching technique regarding ΔE values using different objective measurement methods, including spectrophotometric and digital photographic analyses. The ΔE values decreased with time according to both measurement methods. Therefore, the first null hypothesis that there would be no significant color rebound at different time points was rejected.

Many factors may impact tooth bleaching efficacy and the color relapse, such as the pH and viscosity of the bleaching agent, the concentration of hydrogen peroxide, the contact time of the agent on the tooth surface, the light activation, the number of treatment sessions, the baseline tooth color of the patient, the patient’s age, and the dental and medical history of the patient [10,29]. Hydrogen peroxide is widely used for in-office bleaching treatments at different concentrations [1,2]. The concentration of hydrogen peroxide may influence the number of bleaching agent applications required to obtain the desired bleaching results [30]. Nonetheless, it has been reported that the concentration of 6% hydrogen peroxide promoted effective tooth bleaching similar to the concentration of 37.5% hydrogen peroxide, and there was no influence of hydrogen peroxide concentration on the color rebound after three months following bleaching treatment [12]. The high hydrogen peroxide concentration may cause tooth sensitivity and is avoided using light activation [4,7,29]. The use of a light source for in-office bleaching might ensure a decrease in the working time for bleaching treatment [7,14]. The bleaching gels involve coloring agents or pigments to improve the interaction of visible light, and in doing so, promote the absorption of light and its conversion into heat [7]. The light source influences the bleaching gel by increasing the temperature of the hydrogen peroxide, therefore accelerating the reaction of forming hydroxyl and oxygen radicals [3]. A temperature increase of 10 °C enhances the speed of hydrogen peroxide decomposition by 2.2 times [7]. Previous studies reported that the light activation hastened the in-office bleaching process, but it did not influence the long-term color relapse results [7,8,14].

The color stability of bleached teeth over time is as important as the esthetic results acquired immediately after treatment [8]. A color return may be observed after bleaching treatment, regardless of hydrogen peroxide concentration [5,8,29,31]. It has been reported that a 10% color rebound might occur in the first year of bleaching, and it might increase with time [13,32]. These reports are consistent with the results of this study. However, this color rebound seems to be clinically relevant, since if the value of ΔE is higher than 3.3, the naked eye can distinguish color differences [29]. In the present study, all the ΔE values were higher than 3.3. This color regression might be attributed to the single bleaching treatment sessions, as in previous studies [5,8]. The increase in treatment sessions and the combined use of in-office and at-home treatments may promote better color stability [5,8]. Nonetheless, the increase in the number of sessions might extend the clinical time and cause tooth sensitivity [7]. In-office bleaching treatment may be performed in only one session through light activation [7]. However, it has also been stated that the color rebound after bleaching was not associated with light activation [4,5,7].

In the present study, the color assessment and the calculation of color differences were performed based on the CIELab system. In this system, L* represents the value of lightness or darkness and extends from 0 (black) to 100 (white). a* is the measure of the red–green axis. A positive a* value indicates the red direction, a negative a* value represents the green direction. b* value represents the yellowness–blueness axis. A positive b* value denotes a color in the yellow direction, whereas a negative b* value indicates a color in the blue direction. The color becomes neutral when a* and b* values approach zero [4,5,29]. The value of the L* parameter increases while the values of the parameters a* and b* approach zero after bleaching [29]. In the present study, L* values increased up to 1 week after bleaching, then slightly decreased, which indicates improved luminosity. The parameters of a* and b* values approached zero, which shows an increase in greenness and a decrease in yellowing, respectively. These results are in agreement with previous studies [9,29,33]. However, greater ΔE values were obtained in this study than in previous ones in which the 25% hydrogen peroxide light-activated bleaching technique was used [6,9,10]. This bigger color change might be because the initial color used in previous studies was lighter [29]. It has been reported that better bleaching results were obtained with yellower teeth [34]. The color change relies on the oxidative action of the hydrogen peroxide into the dental organic substrate, dental dehydration, and enamel demineralization. Furthermore, contact time is an important factor for in-office bleaching results [9]. In this study, a standardized application time was used for all patients according to the manufacturer’s instructions. Although the bleaching agent was applied for three cycles in previous studies [6,9,10], the agent was applied four times in the present study. It has been concluded that the same bleaching agent provided greater bleaching efficacy with light activation compared to without light [6]. In the study, great ΔE values were obtained during all test periods after bleaching, which represents effective bleaching results according to the literature [1,7,13,14,15]. The ΔE values are also not enough to discriminate with the human eye. Therefore, it may be said that this bleaching system ensured acceptable whitening during all periods of evaluation.

The evaluation of tooth color after bleaching treatments has great importance [8]. The subjective and objective methods in the measurement of tooth color after bleaching have been evaluated in previous studies, and it has been concluded that the objective methods are more reliable [4,5,8,11,13,29]. One important point of the present study is the comparison of color values measured by two objective methods, including a spectrophotometer and digital photographs. Digital photographic analysis is not a frequently used method in the assessment of color change compared to a spectrophotometer. In this study, the ΔE values obtained by both spectrophotometric and digital photographic analyses decreased with time, but the ΔE values of the spectrophotometer were higher at all test periods. Therefore, the second null hypothesis that there would be no significant difference between color change values evaluated by spectrophotometric and digital photographic analyses was rejected. However, both measuring strategies were able to detect a color change over time. The spectrophotometer might reveal a highly accurate spectral curve indicating the exact L*a*b* values [35]. It has been concluded that the spectrophotometers gave confident and standard results, with 96% accuracy [36]. It has been stated that the use of spectrophotometers is a more effective method in the assessment of color changes over time after bleaching treatment than digital photographs [14] and might show more accurate results regarding tooth color [7,14]. Previous studies have stated that the use of a spectrophotometer seems to be more objective and sensible than digital photographs [6,7,37]. Nevertheless, these studies did not objectively evaluate tooth color by using photographs and software. Therefore, it is hard to say which one gives more accurate results. All photographic methods have limitations since they are dependent on many variables [28]. Taking fully standardized intraoral digital photographs is very difficult, which may lead to misleading results. Both spectrophotometric and digital photographic analyses were able to identify significant ΔE values, and thereby color changes over time, thus the digital photographic analysis method may be used in clinical practice.

The follow-up time used here of 1 week after the end of bleaching treatment is considered to be the minimum time required for color stabilization. In this study, the ΔE values increased for up to 1 week, then reduced. The results acquired immediately after an in-office bleaching session might not be only due to the oxidative action of the hydrogen peroxide in the dental organic substrate [13]. The dental dehydration and enamel demineralization during bleaching may also impact the color [9,13]. The immediate bleaching result may differ from the color measured some weeks later because of rehydration and remineralization [8,9].

Bleaching treatments can affect the quality of life and patients’ psychology [29,34]. Patient perceptions are very important for the assessment of the results of dental treatments [21]. In the present study, all patients stated that they were very satisfied with their teeth color after two years. Nonetheless, the present study has some limitations. Although the sample size was similar to previous studies, it was small, and the participants were young. Only one bleaching product was evaluated. The color measurement could also be performed more frequently, and evaluation periods could be increased. Furthermore, color evaluation with subjective methods is quite hard in clinical practice since several parameters may affect the color, such as daylight, ambient light, ambient conditions, and patient-related factors. Furthermore, it was not taken into account that LED systems lose luminance over time, or that the luminance is dependent on the battery charge.

## 5. Conclusions

From this study, it may be concluded that the in-office bleaching treatment with 25% hydrogen peroxide in combination with photoactivation showed improved bleaching results and maintenance of color stability. Both evaluated measuring methods using spectrophotometric and digital photographic analyses presented significant differences in color change values (ΔE). The quantitative color assessment from the digital photographs using the software may be a promising and useful method to evaluate bleaching results in clinical practice. This method is a highly reproducible method as a permanent database of images may be obtained by digital photographs. Thus, the tooth color may be analyzed at a later date using the same images.

## Figures and Tables

**Table 1:** ΔL, Δa, Δb, and ΔE values acquired by different measurement methods during evaluation periods.

	L* (Mean ± SD)	a* (Mean ± SD)		b* (Mean ± SD)	
Spectrophotometry	70.37 ± 3.44	7.20 ± 1.19		22.90 ± 2.26	
Photography	69.34 ± 4.20	7.01 ± 1.25		22.78 ± 1.99	
	**Δ** **L_1_**	**Δ** **L_2_**	**Δ** **L_3_**	**Δ** **L_4_**	***p* ^†^**
Spectrophotometry	9.63 ± 1.31 ^a^	10.40 ± 1.21 ^b^	7.30 ± 1.20 ^c^	5.21 ± 1.12 ^d^	<0.001
Photography	7.79 ± 0.86 ^a^	9.73 ± 0.90 ^b^	5.95 ± 1.19 ^c^	4.18 ± 1.01 ^d^	<0.001
***p*** **^‡^**	<0.001	<0.001	<0.001	<0.001	
	**Δ** **a_1_**	**Δa_2_**	**Δa_3_**	**Δa_4_**	
Spectrophotometry	−3.79 ± 0.75 ^a^	−3.56 ± 0.76 ^b^	−2.76 ± 0.64 ^c^	−2.05 ± 0.59 ^d^	<0.001
Photography	−3.70 ± 0.71 ^a^	−3.53 ± 0.86 ^b^	−2.81 ± 0.80 ^c^	−2.05 ± 0.70 ^d^	<0.001
***p*** **^‡^**	0.766	0.083	0.005	0.793	
	**Δb_1_**	**Δb_2_**	**Δb_3_**	**Δb_4_**	
Spectrophotometry	−9.61 ± 1.42 ^a^	−9.71 ± 1.24 ^a^	−6.82 ± 1.50 ^b^	−4.73 ± 1.41 ^c^	<0.001
Photography	−8.55 ± 1.67 ^a^	−9.50 ± 1.09 ^b^	−6.59 ± 1.54 ^c^	−4.98 ± 1.43 ^d^	<0.001
***p*** **^‡^**	0.000	0.646	0.276	0.180	
	**ΔE_1_**	**ΔE_2_**	**ΔE_3_**	**ΔE_4_**	
Spectrophotometry	14.22 ± 1.31 ^a^	14.74 ± 1.27 ^b^	10.51 ± 1.06 ^c^	7.51 ± 0.98 ^d^	<0.001
Photography	12.24 ± 1.32 ^a^	14.12 ± 0.91 ^b^	9.46 ± 1.27 ^c^	7.00 ± 1.01 ^d^	<0.001
***p*** **^‡^**	<0.001	<0.001	<0.001	<0.001	

All the calculations were performed considering the measured values at the baseline. _1_: After treatment, _2_: 1 week, _3_: 1 year, _4_: 2 years. Same small letters indicate no statistical difference between the values in the row determined at different times with the same method. *p* ^†^: Significance level among the values measured different times with the same method. *p* ^‡^: Significance level among the values measured with different methods at the same test period.

## Data Availability

Not applicable.
